# Assessment of a Novel Automatic Real-Time PCR Assay on the Cobas 4800 Analyzer as a Screening Platform for Hepatitis C Virus Genotyping in Clinical Practice: Comparison with Massive Sequencing

**DOI:** 10.1128/JCM.01960-16

**Published:** 2017-01-25

**Authors:** Leonardo Nieto-Aponte, Josep Quer, Alicia Ruiz-Ripa, David Tabernero, Carolina Gonzalez, Josep Gregori, Marta Vila, Miriam Asensio, Damir Garcia-Cehic, Gerardo Ruiz, Qian Chen, Laura Ordeig, Meritxell Llorens, Montserrat Saez, Juan I. Esteban, Rafael Esteban, Maria Buti, Tomas Pumarola, Francisco Rodriguez-Frias

**Affiliations:** a Liver Pathology Unit, Departments of Biochemistry and Microbiology, Hospital Universitari Vall d'Hebron (HUVH), Universitat Autònoma de Barcelona (UAB), Barcelona, Spain; b Centro de Investigación Biomédica en Red de Enfermedades Hepáticas y Digestivas (CIBERehd), Instituto de Salud Carlos III, Madrid, Spain; c Viral Hepatitis Laboratory, Liver Unit-Internal Medicine, Vall d'Hebron Research Institute (VHIR), Hospital Universitari Vall d'Hebron (HUVH), Barcelona, Spain; d Roche Diagnostics SL, Sant Cugat del Vallès, Spain; e Liver Unit, Hospital Universitari Vall d'Hebron (HUVH), Universitat Autònoma de Barcelona (UAB), Barcelona, Spain; f Microbiology Department, Hospital Universitari Vall d'Hebron (HUVH), Vall d'Hebron Research Institute (VHIR), Universitat Autònoma de Barcelona (UAB), Barcelona, Spain; Rhode Island Hospital

**Keywords:** diagnostics, genotype identification, hepatitis C virus, molecular subtyping

## Abstract

The unequivocal identification of hepatitis C virus (HCV) subtypes 1a/1b and genotypes 2 to 6 is required for optimizing the effectiveness of interferon-free, direct-acting antiviral therapies. We compared the performance of a new real-time HCV genotyping assay used on the Cobas 4800 system (C4800) with that of high-resolution HCV subtyping (HRCS). In total, 502 samples were used, including 184 samples from chronic HCV patients (from routine laboratory activity during April 2016), 5 stored samples with double HCV genotype infections for testing the limitations of the method, and 313 samples from a screening protocol implemented in our hospital (from May to August 2016) based on the new method to further determine its genotyping accuracy. A total of 282 samples, including 171 from April 2016 (the 13 remaining had too low of a viral load for HRCS), 5 selected with double infections, and 106 from screening, were analyzed by both methods, and 220 were analyzed only by the C4800. The C4800 correctly subtyped 125 of 126 1a/1b samples, and the 1 remaining sample was reported as genotype 1. The C4800 correctly genotyped 38 of 45 non-1a/1b samples (classified by HRCS), and it reported the remaining 7 samples as indeterminate. One hundred two of 106 non-1a/1b genotype samples that were identified using the C4800 for screening were confirmed by HRCS. In the 4 remaining samples, 3 were correctly reported as genotype 1 (without defining the subtype) and 1 was reported as indeterminate. None of the samples were misgenotyped. Four of 7 samples with double HCV infections were correctly genotyped by the C4800. Excluding the 5 selected double-infected samples, the C4800 showed 95.7% concordant results for genotyping HCVs 2 to 6 and 1a/1b subtyping, and 99.2% concordance for subtyping 1a/1b single infections in clinical samples. To improve laboratory workflow, we propose using the C4800 as a first-line test for HCV genotyping and 1a/1b classification, followed by transferring non-1a/1b samples to a center where HRCS is available, if further characterization is needed.

## INTRODUCTION

Hepatitis C virus (HCV) infection is a major global health problem. An estimated 160 to 170 million people are chronically infected with this pathogen worldwide (1–3). Most of these individuals are asymptomatic and unaware of the infection, but some experience liver disease that progresses to fibrosis, cirrhosis, and hepatocellular carcinoma.

The most recent HCV consensus update classifies the virus into 7 confirmed genotypes whose nucleotide sequences differ by >30% to 35% and 67 subtypes with a sequence divergence of 20% to 25% (4, 5). With the current change in the treatment paradigm for this infection, unequivocal HCV subtype 1a and 1b identification before treatment is desired for selecting optimal interferon-free, direct-acting antiviral (DAA) regimens (6), as there are considerable differences in the genetic barriers to DAA resistance between subtypes 1a and 1b (7–14).

The currently available commercial techniques for HCV-1 subtyping fail in 2% to 16% of samples (15–25), because of inaccurate genotyping, a failure to amplify, or a failure to categorize, whereas massive parallel sequencing-based methods, such as the 454 GS Junior ultradeep pyrosequencing platform, provide the most accurate results (10). However, the latter approach requires a highly equipped laboratory with specialized staff.

With the purpose of optimizing the workflow for HCV subtyping in clinical diagnostic laboratories, the aim of this study was to analyze the genotyping/subtyping accuracy of a fast, low-cost assay for use on the recently launched Cobas 4800 system (C4800) (Roche Applied Science, Basel, Switzerland). This assay, which is based on real-time PCR amplification of 3 genomic viral regions (nonstructural protein 5B [NS5B], core, and the 5′ untranslated region [UTR]) and followed by detection with genome-specific probes, was compared with the high-resolution HCV subtyping method (HRCS) (23) routinely used in our liver disease diagnosis unit at University Hospital Vall d'Hebron (Barcelona, Spain).

## RESULTS

The average time needed to complete a run was approximately 2.5 h with the C4800 and 3 days with HRCS. A total of 282 samples were processed by both methods, including 171 from the prospective analysis, 5 selected with double infections, and 106 putative non-1a/1b subtypes from the screening study. Of note, the C4800 reports a result as indeterminate when the HCV control is detected in the 3 reactions, but neither the genotype nor the subtype is identified.

In the prospective study, 126/176 samples were classified by the referral method as genotype 1, 45 as non-1a/1b, and 5 as mixed infections. Of the 126 genotype 1 samples, 125 were correctly subtyped as 1a/1b by the C4800. Only one sample was not subtyped, but it was correctly identified as genotype 1. These data yielded a 99.2% concordance between HRCS and the C4800 (Table 1) for 1a/1b subtyping. Among the 45 non-1a/1b samples, 38 were correctly genotyped (G2 to G5) by the C4800, and 7 were reported as indeterminate.

**TABLE 1:** HCV genotyping and subtyping using a high-resolution method (454 GS Junior platform) and the Cobas 4800 real-time platform

Comparison group (no. of cases)	HRCS (454 GS Junior platform) (no. of cases)	Cobas 4800 system (no. of cases)
HCRS versus Cobas 4800, consecutive samples during April 2016		
Subtype 1a/1b samples (126)	1a (44), 1b (81)	1a (44), 1b (81)
	1b (1)	1 (1)
Non-1 genotype samples (45)	2a (1), 2b (1), 2c (1), 2i (1), 2j (1), 2q (1)	2 (6)
	3a (15), 3b (1)	3 (16)
	4a (2), 4d (12), 4f (1)	4 (15)
	5a (1)	5 (1)
	3k (1), 1l (1), 4d (3), 3a (2)	Indeterminate (7)
Samples with viral loads ≤4 log10 IU/ml (13)	Not analyzed by HRCS due to low viral loads	1a (2), 1b (6), 1 (2), 2 (1), 4 (2)
Selected mixed genotype samples (5)^*a*^	1a (69.2) and 4d (30.7)^*b*^	1a and 4
	1a (96.6) and 4d (3.4)^*b*^	1a and 4
	1b (98) and 1a (2)^*b*^	1b
	1b (97) and 4d (3)^*b*^	1b
	1b (80) and 2b (20)^*b*^	1b
HCV genotype and 1a/1b subtype screening by the C4800, May to August 2016 (313 samples)	Not subtyped by HRCS (207)^*d*^	1a (67), 1b (140)
	1a (2), 1b (1), 3a (1)	1 (3), indeterminate (1)
Non-1a/1b genotyping (102)	2a (5); 2b (2); 2c (8); 2j (2); 2q (2)	2 (19)
	3a (46)	3 (46)
	4a (7); 4d (27); 4f (1)	4 (35)
	1b (84) and 2c (16)^*a*^^,^^*b*^	1b and 2
	1b (86) and 2j (14)^*a*^^,^^*b*^	1b and 2

a Mixed infections.

b Values in parentheses are percentages of sequences (virions) that belong to the given subtype.

^c^ Viral loads of the samples were below the detection limit of the 454 GS Junior platform.

d Samples from the C4800 screening test, when correctly subtyped as 1a and 1b, were not retested by HRCS.

In the 106 putative non-1a/1b samples identified by screening, 102 were correctly genotyped as G2 to G5, including 2 samples with double HCV infections in which the C4800 identified the 2 genotypes. Of the remaining 4 samples, 3 were reported as genotype 1 (without defining the subtype) and 1 was reported as indeterminate.

Thus, the concordance of the C4800 results with those of HRCS for genotyping the single-infected, non-1a/1b prospective and screening samples was 94.6% (140/148) (Table 1). There were no cases of cross-identification between subtypes 1a and 1b, or cross-identification with non-1a/1b genotypes. According to HRCS, the 8 indeterminate samples reported by the C4800 (7 April samples plus 1 screening sample), carried subtypes 3k, 1l, 4d, and 3a.

Seven samples with double HCV infections (5 selected and 2 identified from the screening group) were specifically assayed to investigate the limits of the technique. In 4 of 7 double infections, the C4800 identified the major subtype (1a or 1b) and also the minority genotypes, 2 and 4. In the remaining 3 samples, the major 1b subtype was detected, but not the minority ones (Table 1).

Finally, to describe the genotyping and 1a/1ab subtyping capability of the C4800 in consecutive clinical samples, the 5 selected double-infected samples were excluded from the analysis. The C4800 correctly genotyped 97.1% (269/277) of the clinical samples and it correctly genotyped and 1a/1b subtyped 95.7% (265/277) of them. Furthermore, the method correctly identified 99.2% (125/126) of all 1a/1b subtypes in single infections. Of note, 13 samples with a viral load of ≤4 log10 IU/ml were ultimately run only on the C4800, as this value is below the limit of detection of our HRCS technique (23). Hence, the results in these samples were not confirmed by HRCS and no historical genotyping data to support or refute the C4800 results were available.

## DISCUSSION

Undoubtedly, the most accurate method for HCV subtyping is a high-resolution HCV system based on the phylogenetic analysis of reads obtained by deep sequencing of an NS5B fragment, such as that previously reported in this journal (overall accuracy, 100%) (23). However, not all clinical diagnostic laboratories are equipped with this advanced methodology, and commercial HCV genotyping techniques are reported to have considerable limitations (15–25). In this scenario, we sought a suitable solution for correct HCV genotyping and subtyping in daily laboratory practice.

From the practical viewpoint, the new Cobas 4800 analytical platform tested here is a simple-to-use system that does not require specialized skills. Hence, technicians can easily adapt to its use without a complex training process. As was seen in this study, the system processes decapped primary blood samples directly after centrifugation, as well as aliquoted plasma samples stored at −80°C. The samples were identified by a barcode, and the analyzer was connected online with the laboratory computer system.

The C4800 proved to be a fast (less than 2.5 h per run compared to 3 days for HRCS), easy-to-use, and low-cost system (around 50 euros/sample versus 200 euros/sample for HRCS in our setting) with high specificity for HCV genotyping (correct genotyping of 94.6% of non-1a/1b samples, with no cases of misgenotyping) and 1a/1b subtyping (99.2% correct 1a and 1b classification). Subtypes 1a and 1b account for almost 80% of all HCV infections in Western countries and the United States, and a correct identification of these subtypes is strongly recommended by international societies to optimize DAA-based treatment for chronic HCV infection and to avoid treatment failures (12, 26). Hence, this method could be useful for classifying most of the HCV-infected patients for treatment purposes.

Furthermore, it is likely that the efficacy of DAAs against subtypes other than 1a and 1b is also subtype-dependent (12), which could make correct subtyping relevant for any DAA-based treatment decision. Results from several studies have shown that antiviral efficacy is limited in certain subtypes, especially with ribavirin-free regimens. For example, the efficacy of Viekirax (ombitasvir-paritaprevir/ritonavir) is lower in subtype 4d infections than in other genotype 4 subtypes (27), and the efficacy of Harvoni is lower in subtype 4r infections (28).

At present, the main limitation of the C4800 HCV assay (as well as the other currently available routine HCV genotyping tests) is the failure to assign subtypes other than 1a and 1b. Furthermore, the assay was unable to assign a genotype to samples with subtypes 3k, 1l, 4d, and 3a, which were reported as indeterminate, indicating that there is still room for improvement. However, if the identification of subtypes other than 1a or 1b is required by health providers for clinical decision making, specific primers and probes could be designed and adapted to the system for this purpose.

The C4800 detected some mixed infections in the small number of samples studied. In all cases, the prevalent subtype 1a or 1b was correctly identified, and the minor viral population was detected in 4 of the 7 samples. Additional study is required in HCV double infections to determine whether some genotypes are more easily identified than others in these samples.

In summary, the C4800 properly subtyped virtually all 1a and 1b samples and correctly genotyped the remaining samples with very few indeterminate results and no misclassifications, whereas HRCS subtyped 100% of all the samples. As clinical interest is focused on obtaining the most complete and accurate diagnostic information, we propose combining the rapid technical response of the C4800 with HRCS to improve the HCV-related workflow. In this model, the first-line method would be the C4800 assay for fast genotyping and 1a/1b screening (which fulfills the recommendations of international guidelines in relation to selecting anti-HCV treatment), and the second-line method would be HRCS for subtyping non-1a/1b samples, when needed, and indeterminate C4800 results. Of note, the 454 GS Junior sequencing platform we are currently using will be discontinued in December 2016, but the genotyping/subtyping method developed on this instrument (23) is easily adaptable to any sequencing platform, including single molecule real-time (SMRT) sequencing, without the need for further validation.

This proposed combination of methods would enable initial fast genotyping and optimal classification of 1a and 1b subtypes. Genotype 1 is the most prevalent worldwide and most subtypes are 1a and 1b. In our chronically infected HCV population, subtype 1b accounted for 53.6% and subtype 1a accounted for 22.5% of cases (total, 76.1%) (F Rodriguez-Frias et al., submitted for publication). Therefore, in our setting, only the remaining 24% of samples would require further processing by HRCS, and these results would be reported 3 days later. With preliminary use of this screening protocol from May to August 2016, 106 of 313 samples required further study with HRCS. The observed concordance of the C4800 results with those of HRCS for HCV genotyping in our laboratory was 97%, providing a high degree of confidence for clinical decision making. In addition, the C4800 determines the HCV viral load, which means that the two most important analytical parameters for HCV treatment can be obtained within hours in three quarters of all cases in our setting.

In conclusion, the C4800 assay is a fast, easy-to-use, low-cost technique that shows high specificity for HCV genotyping and 1a/1b subtyping. It cannot assign subtypes other than 1a and 1b, but there were no cases of misgenotyping in our study. In addition, the system detected some mixed infections, but the sensitivity for this purpose seems to be associated with the composition of the genotype mixture. Further studies are required to establish the sensitivity for different genotypes present as minor components of mixed infections and to determine the accuracy of the method in samples with very low viral loads.

## MATERIALS AND METHODS

### Patients.

A total of 502 samples were included in the study. Plasma samples from 184 consecutive HCV-infected patients were collected during April 2016 (i.e., the entire month's routine HCV-related workload), and stored frozen at −80°C until they were processed by the C4800 and HRCS. Thirteen of the 184 samples were ultimately run only on the C4800, as the viral loads of these samples were too low for HRCS testing. To study the limitations of the method for identifying mixed infections, we selected 5 samples from our blood serum bank that were diagnosed with double HCV infections. In addition, to increase the number of non-1a/1b samples and further test the method's genotyping capability, we analyzed 313 samples from a screening protocol implemented in our hospital using the new technique (from May to August 2016). Of these 313 samples, 106 identified as genotypes other than 1a/1b by the C4800 were tested by HRCS to confirm the results. In summary, of the 502 samples studied, 282 were tested by both methods, including 171 from routine activity during April 2016, 5 selected mixed infections, and 106 non-1a/1b subtypes from the screening protocol. The remaining 220 samples, including 207 genotype 1a/1b from screening and 13 with viral loads ≤4 log10 IU/ml, the detection limit of HRCS, were tested only by the C4800 (Table 1).

Viral loads were measured in all samples by real-time PCR (RT-PCR) on a Cobas 6800 system (Roche Applied Science, Basel, Switzerland). The median HCV RNA level was 6.32 log10 IU/ml (range, 2.3 to 6.8 log10 IU/ml). In all analyses, the technicians analyzing the samples were unaware of the patient-associated demographic and clinical information.

### High-resolution HCV subtyping.

The high-resolution method described in our previous report (23), which has an overall HCV subtyping accuracy of 100% in samples with a viral load ≥4 log10 IU/ml, was used as the comparison reference for this study. The method is based on the phylogenetic analysis of the NS5B fragment of the HCV genome, as recommended by Simmonds et al. (4). Briefly, HCV-RNA was extracted with the Cobas AmpliPrep system (Roche Diagnostics, West Sussex, United Kingdom) using the total nucleic acid isolation (TNAI) kit. Nested RT-PCR amplification of 20 samples per run was performed using NS5B-specific primers in a multiplex analysis. Each patient was labeled using Roche's validated multiplex identifier (MID). Massive parallel sequencing was carried out on the 454 GS Junior platform, obtaining at least 1000 sequences (reads) per patient sample. Data were analyzed using an in-house developed R-based program.

### RT-PCR-based genotyping on the Cobas 4800 system.

The HCV genotype/subtype assay evaluated here is based on an RT-PCR technique that uses 3 different sets of primers to amplify the 5′ UTR, core, and NS5B regions. It comprises 2 modules. The first, named the x480 Instrument, is a fully automated sample preparation and RNA extraction unit. It uses primary vials and requires 400 μl of plasma and a specific reagent, which are dispensed in a 96-well plate in less than 20 min of setup time. The plate is then manually transferred to the second module, named the z480 Analyzer, which carries out RT-PCR amplification and detection using fluorescently labeled oligonucleotide probes specific for HCV genotypes 1 to 6 and for subtypes 1a and 1b with internal positive/negative controls; genotypes 2, 3, and 6 are determined in the 5′ UTR; genotypes 1, 4, and 5 are determined in the core gene; and subtypes 1a and 1b are determined in the NS5B region.
